# Effectiveness of a Telemedicine Exercise Program to Improve Lung Function in Young Adults After COVID-19: A Pilot Study

**DOI:** 10.3390/healthcare14060718

**Published:** 2026-03-11

**Authors:** Eyckle C. H. Wong, Raymond W. M. Lo, Rachel L. C. Kwan, Natalie N. M. Chan, Sara W. Y. Lam, Ruby Y. K. Ng, Suyi K. C. Wong, Grace P. Y. Szeto

**Affiliations:** School of Medical and Health Sciences, Tung Wah College, Hong Kong SAR, China; raymondlo@twc.edu.hk (R.W.M.L.); rachelkwan@twc.edu.hk (R.L.C.K.); 20002396@twc.edu.hk (N.N.M.C.); 20002411@twc.edu.hk (S.W.Y.L.); 20002395@twc.edu.hk (R.Y.K.N.); 20002369@twc.edu.hk (S.K.C.W.); graceszeto@twc.edu.hk (G.P.Y.S.)

**Keywords:** telemedicine, exercise therapy, respiratory function tests, COVID-19

## Abstract

**Background**: COVID-19 can have adverse effects on individuals’ lung functions for up to 6 months or more after the episode. As a result, people may be reluctant to exercise, and this can have further adverse effects on their lung capacity and fitness. This study aimed to examine the effectiveness of a telemedicine program designed to increase the exercise participation of young adults after COVID-19 and evaluate the changes in lung function after exercise training. **Methods**: The quasi-experimental pre–post study recruited sixty university students who had suffered from COVID-19 within the past 12 months. Four pulmonary outcomes were compared: forced expiratory volume in one second (FEV1), forced vital capacity (FVC), peak expiratory flow rate (PEFR), and the ratio of FEV1 to FVC. The telemedicine exercise (TE) group (*n* = 36) received an intervention to carry out regular stepping exercise (up to 10,000 steps) via online video instruction and frequent WhatsApp reminder messages. The control group (*n* = 24) only received an initial WhatsApp message to carry out regular stepping exercise, with no further follow-up. **Results**: The FVC, FEV1, and FEV1/FVC ratio revealed significant overall improvement both within groups and between groups (*p* < 0.001), with moderate effect sizes. PEFR showed a significant improvement within groups (*p* = 0.007) but not between groups (*p* = 0.533). The TE group recorded a significant increase in daily step count (from 7165 to 9733, *p* < 0.001) after 4 weeks of training. The control group showed a significant reduction in step count (from 6975 to 6442, *p* = 0.049). **Conclusions**: The results confirmed the beneficial effects of the telemedicine exercise program in contributing to increased exercise participation and improved lung functions.

## 1. Introduction

The impact of COVID-19 has been shown to severely compromise respiratory function in affected patients. The virus can infiltrate both the upper and lower respiratory tracts, causing significant inflammation in the alveoli of the lungs [1,2,3]. This condition may lead to a marked decline in pulmonary function, particularly if patients do not receive timely and appropriate medical intervention [1,3]. The respiratory system struggles to intake oxygen and expel carbon dioxide, resulting in clinical signs such as shortness of breath, persistent cough, sputum retention, and impaired daily mobility. Unresolved COVID-19 conditions, often referred to as “long COVID,” are characterized by symptoms such as persistent cough and sputum production lasting beyond six months [4,5].

Evidence suggests that regular exercise can help reverse the symptoms and conditions associated with COVID-19 [6,7,8,9]. Physical activity programs can counteract muscle atrophy and fatigue observed in patients with long COVID [10,11,12]. Different studies have used various forms of exercise such as aerobic exercise, cycling, walking, and stepping in pulmonary rehabilitation programs with good success in improving lung function and exercise tolerance [6,7,8]. During the COVID-19 pandemic, many countries imposed restrictions on social interactions, which accelerated the development of telemedicine programs to meet the demand for managing COVID-19 patients with deteriorating cardiopulmonary conditions at home [10,11,12]. According to the local regulations in Hong Kong, many people stayed home and could not attend normal work or social activities. As a result, many hospitals and clinics developed telemedicine services, incorporating home-based exercise regimes monitored through a telemedicine system. These initiatives marked a new era in rehabilitation which has continued to develop even well after the global COVID-19 pandemic has settled. With rapid advances in digital technology, many countries have continued to develop these remote health monitoring services as a more cost-effective model for health care delivery, even after the end of the pandemic [13,14]. The benefits of videoconferencing interventions include the concurrent management of many patients and reduced numbers of patients travelling to clinical centers. Some countries have established national frameworks and guidelines for developing telemedicine for cardiovascular diseases [14,15]. Telemedicine services can be designed to achieve objectives comparable to those in rehabilitation centers by ensuring and optimizing continuity of care through home-based accessible interventions. Telemedicine programs can tailor rehabilitation programs and educational strategies to facilitate self-management and lifestyle changes [16]. The downside would be the lack of direct in-person instant feedback and guidance that can be provided when patients attend therapy in the clinical setting [17]. While some systematic reviews have indicated that telemedicine exercise services can be as feasible and efficient as conventional physiotherapy in terms of functionality level and quality of life, there are indeed many limitations and challenges in this remote form of intervention [18]. These include the difficulty in ensuring proper exercise technique, and the lack of direct detailed examination of the patient’s physical condition. There may be false impressions or miscommunication on both sides, and it is also more difficult to form an appropriate therapist–patient relationship. Therapists not only need to provide proper instructions to patients but must also motivate them to continue exercising [15,17]. Assessing patients’ conditions properly, providing suitable and individualized interventions, and sustaining patients’ motivation are all important issues to ensure the effectiveness of telemedicine programs.

In response to the COVID-19 pandemic, governmental and non-profit organizations in Hong Kong have piloted telemedicine services aimed at reducing patients’ travel times and optimizing physical well-being [10,12]. These initiatives encompass three main domains: telemedicine and teleconsultation provided by medical professionals, telemonitoring of patients’ clinical data to aid in medical feedback and intervention, and tele-support through online services offering information and advice to patients and caregivers. These initiatives are integrated into the “HA Go” app, which was originally designed to enable patients to make appointments for medical consultations in public hospitals and clinics [19]. Following the outbreak of COVID-19, the Hospital Authority in Hong Kong expanded the use of telemedicine technologies, incorporating video conferencing and mobile apps to facilitate treatment follow-ups and support rehabilitation services, including online exercise instructions for patients with various chronic diseases.

The application of telemedicine programs in Hong Kong is still in the developmental stage, and their clinical effectiveness across specific patient populations has not yet been clearly established. This study aimed to compare the effects of a 4-week telemedicine exercise program on lung function outcomes and participants’ adherence to a structured stepping regime. It is hypothesized that frequent reminders and clear instructions from a video will enhance motivation in the participants in the telemedicine group compared with the control group, leading to greater improvements in pulmonary function.

## 2. Methods and Materials

### 2.1. Study Design

This is a quasi-experimental pre–post study design with two groups. The blinding of participants and therapists was not feasible in this study, as participants knew if they were participating in the telemedicine exercises versus the control group. Ethical approval was obtained from Tung Wah College (No: MHS/PT/AY23-34/1) prior to the study’s commencement. The data was collected from September 2023 to March 2025.

### 2.2. Participants

Undergraduate students who had suffered at least 1 episode of COVID-19 within the last 12 months and with long COVID symptoms were recruited (aged between 18 and 30). Posters were displayed on campus, and students were recruited by convenience sampling. Inclusion criteria were good general health, medical stability, being free from acute illness, and lack of chronic diseases such as diabetes or hypertension. Exclusion criteria were smoking history, serious illness, especially in the cardiorespiratory system, or any previous serious traumatic injury affecting the spine, the heart, or the lungs.

The potential participants were screened using the 2020 English version of the Physical Activity Readiness Questionnaire (PAR-Q), and only those who answered “No” to all questions were included. For lung function assessment, in addition to the standard spirometry values, the Global Lung Function Initiative equation [17] was used to exclude abnormal data after obtaining lung function test results. The American Thoracic Society and the European Respiratory Society recommend that the bottom 5th percentile (z-scores ≤ 1.645) be defined as the lower limit of normal. Z-scores of the ratios of forced expiratory volume to forced vital capacity (FEV1/FVC) and forced vital capacity (FVC) values smaller than −1.645 were considered unusually low and excluded from the analysis of this study.

### 2.3. Telemedicine Exercise Intervention and Control Groups

Participants in the telemedicine exercise group received instructions about the four-week telemedicine exercise program. It was a daily 10,000-step exercise program based on a systematic review on steps and health outcomes [20]. The 10,000 steps campaign for a healthy life was developed by the Department of Health and the Hong Kong Government in 2023/24 [21]. In our study, for the safe and more easily adherent 10,000-step program, an exercise video was created to support the motivation of the clients. The exercise video was delivered to all participants through WhatsApp (Version 2.23.21) and uploaded to the webpage “tung-wah.odoo.com,” with a QR code provided. They were instructed to perform the exercises at least 5 times per week. The tele-exercise was essentially a 30 min stepping exercise at a rate of 120 bpm. It corresponded to 4.8 METs to reach the moderate intensity level for training according to ACSM standards [22]. The 120 bpm rate of the step exercise was useful for predicting intensity aligned with age-associated differences in physiological response to perceived exercise of moderate and/or vigorous intensity [20,22].

Participants in the TE group received constant reminders via WhatsApp concerning the 10,000 steps program [23]. Concurrently, Google or Apple pedometer applications were installed on their mobile devices to ensure consistent stepping rhythm. The validation of step-count applications was studied, and step errors were found to be below 3% [22,24]. The website offered live tele-conferencing with the research team to address any difficulties in engaging with the telemedicine exercise program. The participants were instructed on the use of the Google pedometer “app” (Version 8.1), which was used to keep records of step counts, and these were reported weekly to the research team through WhatsApp messages.

The control group only received the message about the “10,000 step exercise” and the benefits of step exercise at the start of the four-week program. They did not receive any further information on the exercise program app or any soft reminders via WhatsApp message to carry out the stepping exercise. They were still required to send back the step count reports to the research team on a weekly basis.

### 2.4. Outcome Measures

Demographic information was collected via self-reported questionnaires. Basic body measurements, including weight, height, and body mass index (BMI), were obtained using the InBody-770 device (InBody, Seoul, Republic of Korea). The primary outcomes measured included the average daily step count, tracked by a pedometer application on participants’ mobile phones, and changes in lung function measures pre- and post-intervention.

Lung function tests were conducted using the Scientech Spirolab™ device (Medical International Research (MIR) USA Inc., New Berlin, WI, USA). Calibration and reliability procedures for the lung function machine were performed according to the standard of the American Thoracic Society daily before the assessment. Assessments were performed both before and after a four-week intervention to evaluate improvements in lung function through spirometry, as well as participants’ reported experiences of dyspnea during daily activities.

During the assessment, four pulmonary outcomes were measured: forced expiratory volume in one second (FEV1), forced vital capacity (FVC), peak expiratory flow rate (PEFR), and the ratio of FEV1 to FVC. The FVC measurement indicates the total amount of air a person can forcibly and quickly exhale after taking a deep breath, while FEV1 reflects the amount of air exhaled in one second during the FVC test. The FVC and FEV1 ratios are commonly used in identifying specific types of lung disease.

The measurement units for the pulmonary outcomes were FVC in liters, FEV1 in liters, FEV1/FVC as a percentage, and PEFR in liters per minute. The best values from three acceptable maneuvers with consistent FVC and FEV1 results were used for analysis.

To monitor compliance, participants were also provided with hard copies of logbooks to record whether they completed the moderate intensity stepping exercise each day.

### 2.5. Data Analysis

Data were analyzed utilizing the Statistical Package for the Social Sciences (SPSS) version 29.0 (IBM, Armonk, NY, USA). An independent *t*-test and chi-square test were used to compare the demographic data between the telemedicine exercise and control groups. A two-way repeated measures ANOVA was conducted to evaluate the changes before and after the 4-week telemedicine exercise program, encompassing average daily step counts and values from lung function tests (FVC, FEV1 and PEFR) between groups. Potential confounders, including baseline differences, were included in the ANCOVA model when appropriate. Subsequent between-group and within-group comparisons were performed with Bonferroni correction. Data were analyzed with a *p*-value < 0.05 adopted as the level of significance.

## 3. Results

### 3.1. Demographic Information

Sixty university students, 26 males and 34 females, were included in the study. They were randomly allocated to either the telemedicine exercise (TE) group or the control group. The age of the two groups showed a statistically significant difference (*p* = 0.026), while the weight and BMI values were comparable between the groups (weight: *p* = 0.313; BMI: *p* = 0.330) (Table 1).

### 3.2. Lung Function Outcomes

Baseline measurements of lung function were compared between telemedicine exercise and control groups before intervention (Table 2). There were no statistical differences in the FVC and FEV1 between the two groups (both *p* < 0.05). As there was statistical significance in terms of FEV1/FVC between the two groups before intervention, subsequent analyses regarding FEV1/FVC were adjusted using baseline measurements as covariates.

After completing the 4-week 10,000-step exercise program, all participants in the TE group showed significant changes in lung function parameters, including FVC, FEV1, and FEV1/FVC. In contrast, there were no significant changes in these lung function parameters in the control group. The results are summarized in Table 3.

For FVC, the between-group analysis showed a *p*-value of 0.429 for FVC pre-treatment and 0.753 post-treatment, indicating no significant difference between the two groups. Within-group analysis showed a significant improvement in FVC for the telemedicine exercise group (*p* < 0.001 pre-treatment, *p* = 0.143 post-treatment) compared to the control group. The partial eta squared values indicated a moderate effect size for FVC improvement in the TE group (0.324 pre-treatment, 0.037 post-treatment). The overall within-group analysis indicated a significant improvement in FVC for both groups (*p* < 0.001, partial eta squared = 0.257), while the between-group analysis showed a significant difference in FVC values (*p* = 0.033, partial eta squared = 0.076). The group x time interaction *p*-value of 0.575 with a partial eta squared of 0.005 suggests no significant interaction effect between group and time for FVC values. Overall, the TE group showed a significant improvement in FVC compared to the control group, with a moderate effect size.

For FEV1 before the intervention, the average FEV1 for both groups was similar for the telemedicine group and the control group (3.47 ± 0.76 L and 3.47 ± 0.74 L, respectively), with no significant difference between the groups (*p* = 0.988). After the intervention, the telemedicine group showed an improvement in FEV1 to 3.74 ± 0.75 L, while the control group had a smaller increase to 3.50 ± 0.76 L. However, the difference in the post-intervention FEV1 between the groups was not statistically significant (*p* = 0.229). When looking at the within-group analysis, both groups showed a significant improvement in FEV1 from pre- to post-intervention, with the telemedicine group having a larger effect size (ȵ^2^ = 0.650) compared to the control group (ȵ^2^ = 0.013). Overall, both within-group and between-group comparisons were statistically significant (*p* < 0.001), with larger effect sizes for the telemedicine group. The Group x Time interaction was not significant (*p* = 0.547, ȵ^2^ = 0.006), indicating that the changes in FEV1 were similar between the two groups over time.

The results for FEV1/FVC showed that there was a significant improvement in the FEV1/FVC ratio in the TE group, with a post-exercise ratio of 90.26 compared to the control group’s 85.63. The between-group analysis indicated a *p*-value of less than 0.001, with a partial eta squared value of 0.315, showing a significant difference between the two groups. Additionally, there was a significant within-group improvement in both groups. The overall within-group *p*-value was 0.001, with a partial eta squared value of 0.170, while the overall between-group *p*-value was less than 0.001, with a partial eta squared value of 0.315. The group x time interaction had a *p*-value of 0.315 and a partial eta squared value of 0.315. The covariates in the model were evaluated using an FEV1/FVC(Pre) value of 87.62. Overall, the telemedicine exercise intervention showed a significant improvement in lung function compared to the control group.

For PERF, the within-group analysis showed a non-significant improvement for both groups, with a *p*-value of 0.205 for the telemedicine group and 0.842 for the control group. The partial eta squared values for the within-group analysis were 0.028 for the TE group and 0.001 for the control group. Overall, there was a significant improvement in PEFR post-exercise for both groups, with an overall within-group *p*-value of 0.007 and a partial eta squared value of 0.120. The group x time interaction was also significant, with a *p*-value of 0.007 and partial eta squared value of 0.007.

### 3.3. Average Daily Steps in the Telemedicine Exercise and Control Groups

In the TE group, the average step count was 7165 ± 1255 at baseline and increased to 9733 ± 1531 after four weeks of training (Table 4). The exercise program set the target at “10,000 steps” and the average steps completed by the participants were 9733, which means that the goal was achieved by more than 50% of the participants in this group. Conversely, the control group exhibited an initial average of 6975 ± 2022, which declined to 6442 ± 1865. The difference in steps achieved by the two groups led to a significant between-group difference in *p* < 0.001.

## 4. Discussion

Apart from manifestations such as fatigue, sleep disturbances, and cognitive impairment, young adults afflicted with COVID-19 subjectively experience a notable decline in pulmonary function, characterized by a reduction in FVC and FEV1, alongside a compromised diffusion capacity. These alterations contribute to ineffective respiration and a diminished overall lung capacity [4]. The deconditioning of the diaphragm and intercostal muscles following COVID-19 attack significantly impacts the efficiency of breathing, which could have adverse effects on a number of different pulmonary functions [25,26].

The four-week telemedicine exercise program produced positive outcomes in FVC and FEV1, caused by the high number of daily average steps achieved. The exercise increased the pulmonary capacity of the participants. The daily step goal enhanced exercise habits and transformed sedentary behaviors into active engagement in aerobic capacity training. These results are consistent with those reported by previous research involving in-person physical exercise training programs. In the systematic review by Rahmati et al. [12], 10 studies reported significant improvements in FVC and FEV1 after a physical rehabilitation program in post-discharge COVID-19 patients. The present study was able to produce significant improvement in FVC and FEV1 via a telemedicine program without the direct face-to-face contact of therapists with patients, and this provides important evidence to support the effectiveness of telemedicine service models [13,14,15,17,18].

The rhythmic step exercises acted as a training stimulus for the diaphragm, the intercostal muscles, and accessory muscles in respiration. This can have positive effects on muscle hypertrophy and enhanced neuromuscular efficiency. The stronger the inspiration, the deeper the total volume of air that can be exhaled (FVC). The stronger the expiration, the stronger FEV1 exhaled with good expiratory muscle function [2,3]. The improvement enhanced the respiratory muscles and resulted in less fatigue during aerobic exercise in training for ventilation. The stretching effect on the lung tissue and chest wall can also improve the work of breathing as the changes in FVC and FEV1 improved lung compliance [27].

During the stepping exercise, cardiac output and stroke volume improved. Perfusion to the pulmonary capillaries increased [28]. The indirect effect of the change in FVC and FEV1 showed that the efficiency of the lung function contributed to the matching of the ventilation and perfusion ratio in the improvement of FVC and FEV1.

The participants in the present study were mainly young healthy adults prior to suffering COVID-19. Hence, their values in FEV1 to FVC were in the normal range, and therefore the absolute change in their values was relatively small after exercise training. If the clients had respiratory illnesses such as asthma or obstructive lung disease, their baseline FEV1 to FVC ratio would be in the clinical range and the changes would need to be of a greater extent in order to restore their lung function [25,26].

The present telemedicine exercise program, with the video of rhythmic step exercises to be performed daily, maintained an approximate level of 4–5 METs for pulmonary exercise training [29]. This is the appropriate exercise intensity for young adults to improve their respiratory fitness. Moderate-intensity exercises have been reported to produce cardiovascular and respiratory benefits enhancing oxygen delivery and utilization [23]. While the present telemedicine training program mainly focuses on stepping exercise, eventually the participants may feel well enough to attempt other forms of exercise. This can have a long-term benefit on their fitness and well-being in general.

The telemedicine exercise program demonstrated feasibility to increase participants’ personal drive to undertake this volume of physical activity. In contrast, the control group demonstrated a decline in the step count, and this confirms the importance of having appropriate guidance and facilitation to sustain the motivation of the individuals to continue exercising on their own. This result is consistent with other research findings supporting the benefits of adopting a telemedicine exercise program, which yielded similar benefits for individuals with cancer, chronic pain syndromes, and among the aging population in community settings [13,16,18].

The telemedicine exercise program allowed for a flexible schedule and enabled the participants to perform exercises at home daily. The positive reinforcement with the video and the soft reminders over WhatsApp conveyed the importance of the daily aerobic exercise. This reduced the dropout common in the rehabilitation process. With the use of pedometer apps, the steps can be tracked automatically. Past research has also demonstrated the validity of step-counting “apps” to monitor the exercise intensity of participants [24,30]. This element may have enhanced the performance of the participants and serves as a motivator to achieve the goal [22,31].

The telemedicine exercise program has demonstrated its significant role in enhancing the respiratory system and serves as a vital component for pulmonary rehabilitation following hospital discharge, ultimately promoting pulmonary health [6]. The current telemedicine exercise program has the potential to be included in the Hong Kong healthcare system under the use of the HA GO “app”. While there is a global trend of continuing the development of telemedicine interventions due to advances in technology and cost-effectiveness issues, it is often difficult to sustain the long-term motivation of individuals to comply with regular exercise training. Various research approaches are emerging to find solutions for these issues, including the use of gamification, setting up personalized reminders or other ways to increase the accessibility and flexibility of exercise interventions.

This program aims to reduce patients’ travel time and optimize physical well-being in community settings. The 10,000 steps telemedicine exercise program also demonstrated a safe and effective adherence method for improving lung function. Given the constraints of the present study, despite significant changes in the lung functions, the results can only be generalized to young healthy participants. The participants recruited may also be in different stages of recovery after COVID-19, and their responses to exercise training would be different; this is a practical limitation of the present study. We also acknowledge the risk of selection bias caused by using convenience sampling. Subsequent studies should be conducted as randomized controlled trials, with increased sample size and longer intervention and follow-up periods. Recruiting a more homogeneous group of participants would also enhance the validity of the present study. The implementation of a more precise and highly accurate pedometer app may yield a more scientifically robust assessment of the 10,000 steps telemedicine exercise program. In addition, further clinical investigations involving cases with significant pulmonary impairment may further elucidate the substantial importance of this program.

## 5. Conclusions

The telemedicine exercise program has the potential to become a vital component of pulmonary rehabilitation post-COVID-19, effectively combining physical recovery with psychological support. The present study demonstrated the effectiveness of the telemedicine exercise program, which produced positive engagement in the participants. Significant improvement in lung function was achieved. Continued research on the use of telemedicine exercise is necessary to refine program delivery and establish long-term outcomes for lung function recovery. The adoption of different forms of exercise in addition to stepping will enhance the participation and motivation of patients. Different applications of this technology can also be explored further. Many potential areas can be reviewed to improve the efficacy of telemedicine services. Adopting telemedicine services can enhance the effectiveness of rehabilitation following COVID-19 and for other chronic disease patients in the community.

## Figures and Tables

**Table 1:** Demographic characteristics of the subjects in the telemedicine exercise and control groups.

	Telemedicine Exercise (*n* = 36)	Control (*n* = 24)	Between-Group *p*-Value
Gender (number of male/female)	14/22	12/12	0.395
Age (years)	22.47 ± 2.67	24.25 ± 3.10	* 0.026
Weight (kg)	61.94 ± 8.03	64.00 ± 7.13	0.313
BMI (kg/m^2^)	22.36 ± 1.76	23.06 ± 3.20	0.330

Data are expressed as ‘mean ± standard deviation’ unless otherwise stated. * *p*-value < 0.05.

**Table 2:** Baseline measurement in lung function parameters between the telemedicine exercise and control groups.

	Telemedicine Exercise (*n* = 36)	Control (*n* = 24)	Between-Group *p*-Value
FVC (L)	3.90 ± 0.85	4.09 ± 1.02	0.429
FEV1 (L)	3.47 ± 0.76	3.47 ± 0.74	0.988
FEV1/FVC (%)	88.96 ± 5.14	85.61 ± 4.81	* 0.014

* *p*-value < 0.05. Data are expressed as ‘mean ± standard deviation’ unless otherwise stated.

**Table 3:** Lung function parameters between the telemedicine exercise and control groups pre- and post-training.

Outcome Measures	Within-Group Differences								Between-Group Differences	
	Telemedicine Exercise (*n* = 36)				Control (*n* = 24)				Partial Eta Squared (ȵ^2^)	*p*-Value
	Baseline Mean (SD)	Post-Intervention Mean (SD)	Partial Eta Squared (ȵ^2^)	*p*-Value	Baseline Mean (SD)	Post-Intervention Mean (SD)	Partial Eta Squared (ȵ^2^)	*p*-Value		
FVC (L)	3.90 ± 0.85	4.08 ± 0.83	0.324	* <0.001	4.09 ± 1.02	4.15 ± 0.97	0.037	0.143	0.076	* 0.033
FEV1 (L)	3.47 ± 0.76	3.74 ± 0.75	0.650	* <0.001	3.47 ± 0.74	3.50 ± 0.76	0.013	0.395	0.375	* 0.001
FEV1/FVC (%) ^	87.62 ± 0.00	90.26 ± 0.56	0.284	* <0.001	87.62 ± 0.00	85.63 ± 0.69	0.128	* 0.005	0.315	* <0.001
PEFR (L/min) ^+^	6.69 ± 0.00	6.92 ± 0.18	0.028	0.205	6.69 ± 0.00	6.73 ± 0.23	0.001	0.842	0.007	0.533

Data are expressed as ‘mean ± standard deviation’ unless otherwise stated. * *p*-value < 0.05. Two-way repeated measures ANOVA with Bonferroni correction was used. Covariates appearing in the model are evaluated using ^ FEV1/FVC(Pre) = 87.62 and ^+^ PEFR(Pre) = 6.69.

**Table 4:** Average daily steps between the telemedicine exercise and control groups pre- and post-training.

Outcome Measures	Within-Group Differences								Between-Group Differences	
	Telemedicine Exercise (*n* = 36)				Control (*n* = 24)				Partial Eta Squared (ȵ^2^)	*p*-Value
	Baseline Mean (SD)	Post-Intervention Mean (SD)	Partial Eta Squared (ȵ^2^)	*p*-Value	Baseline Mean (SD)	Post-Intervention Mean (SD)	Partial Eta Squared (ȵ^2^)	*p*-Value		
Average daily steps (*n*)	7164.69 ± 1255.18	9733.13 ± 1531.14	0.709	* <0.001	6975.00 ± 2021.68	6441.67 ± 1865.22	0.065	* 0.049	0.587	* <0.001

Data are expressed as ‘mean ± standard deviation’ unless otherwise stated. * *p*-value < 0.05.

## Data Availability

The original contributions presented in this study are included in the article. Further inquiries can be directed to the corresponding authors.

## Abbreviations

The following abbreviations are used in this manuscript:

PAR-Q	Physical Activity Readiness Questionnaire
FEV1/FVC	Ratio of forced expiratory volume in 1 s to forced vital capacity
FVC	Forced vital capacity
FEV1	Forced expiratory volume in one second
PEFR	Peak expiratory flow rate
BMI	Body mass index
SPSS	Statistical Package for the Social Sciences
